# L-arginine-containing mesoporous silica nanoparticles embedded in dental adhesive (Arg@MSN@DAdh) for targeting cariogenic bacteria

**DOI:** 10.1186/s12951-022-01714-0

**Published:** 2022-12-01

**Authors:** Marta López-Ruiz, Francisco Navas, Paloma Fernández-García, Samuel Martínez-Erro, Mª Victoria Fuentes, Isabel Giráldez, Laura Ceballos, Carmen Mª Ferrer-Luque, Matilde Ruiz-Linares, Victoria Morales, Raúl Sanz, Rafael A. García-Muñoz

**Affiliations:** 1 Faculty of Health Sciences, IDIBO Research Group, Rey Juan Carlos University, Madrid, Spain; 2 Department of Stomatology, School of Dentistry, University of Granada, Campus de Cartuja, Colegio Máximo S/N, 18071 Granada, Spain; 3 Department of Chemical and Environmental Technology, Rey Juan Carlos University, C/ Tulipán S/N Móstoles, 28933 Madrid, Spain

**Keywords:** Dental caries, Dental adhesive, Drug delivery systems, Mesoporous silica nanoparticles, L-arginine

## Abstract

Dental caries is the major biofilm-mediated oral disease in the world. The main treatment to restore caries lesions consists of the use of adhesive resin composites due to their good properties. However, the progressive degradation of the adhesive in the medium term makes possible the proliferation of cariogenic bacteria allowing secondary caries to emerge. In this study, a dental adhesive incorporating a drug delivery system based on L-arginine-containing mesoporous silica nanoparticles (MSNs) was used to release this essential amino acid as a source of basicity to neutralize the harmful acidic conditions that mediate the development of dental secondary caries. The in vitro and bacterial culture experiments proved that L-arginine was released in a sustained way from MSNs and diffused out from the dental adhesive, effectively contributing to the reduction of the bacterial strains *Streptococcus mutans* and *Lactobacillus casei*. Furthermore, the mechanical and bonding properties of the dental adhesive did not change significantly after the incorporation of L-arginine-containing MSNs. These results are yielding glimmers of promise for the cost-effective prevention of secondary caries.

**Graphical Abstract:**

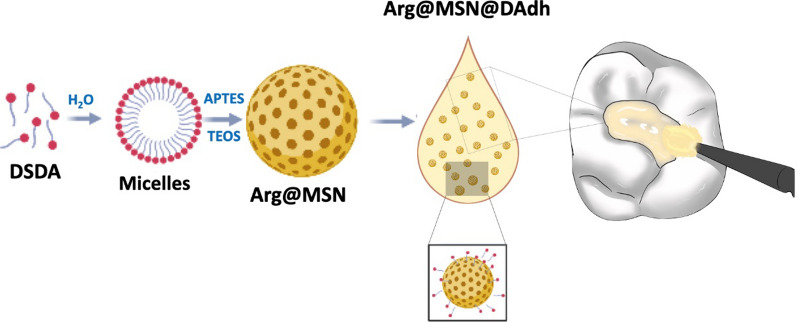

**Supplementary Information:**

The online version contains supplementary material available at 10.1186/s12951-022-01714-0.

## Introduction

Globally, dental caries remains the most prevalent biofilm-mediated oral disease affecting the permanent teeth of 2.3 billion people worldwide [1]. Resin composites are the first choice and most widely used material to restore caries lesions and defective restorations in anterior and posterior teeth [2, 3]. Several reasons contribute to their popularity, such as their high mechanical and aesthetic performance, easy handling, and compatibility with the principles of Minimal Intervention Dentistry, as they are adhesively placed and therefore require less invasive preparations [4, 5]. However, the longevity of resin composite restorations is limited, with secondary caries at restoration margins due to biofilm acids being one of the main reasons for clinical failure [4–7]. Secondary caries have been related to the presence of marginal defects in a restoration due to the polymerization shrinkage of adhesive and composite resins, which prompts the degradation of the hybrid layer, allowing cariogenic bacteria penetration and proliferation, and further degradation of the adhesive, establishing a vicious cycle [2, 8]. Additionally, secondary caries is significantly more prevalent in high caries risk patients [6]. This is of paramount relevance, as resinous components may upregulate the growth of acidogenic bacteria by providing a source of carbon and oxygen, which implies that the biofilm is not modulated and causes the caries lesion to develop again [9, 10].

One of the strategies to prevent the development of secondary caries is to introduce biofilm modifiers, mainly in dental adhesives, since they inhibit microbial populations with cariogenic action and enhance the growth of pro-healthy bacteria [11–22]. However, the disadvantage of the free addition of antibacterial agents or drugs into resin composites is the short duration of the antibacterial effect due to the absence of restrictions to be released, as well as the possible impairment of the mechanical and adhesive properties [23, 24].

Therefore, the release of drugs using metallic nanoparticles, polymeric particles, micelles, liposomes, and mesoporous silica nanoparticles (MSNs) has been widely explored in dentistry [25–27]. In comparison with other nanoparticles, MSNs have the unique characteristics of mesoporous structure, large surface area and pore volume, stable physicochemical properties, easy surface modification and good biocompatibility [28–32]. MSNs could therefore be promising materials for the incorporation of antimicrobial drugs into resin composites due to their proven biocompatibility, similar to silica particulate fillers used in ordinary adhesives, and the large surface area and pore volume that would allow exceptional loading capacity [33]. In the typical synthesis of MSNs, a structure-directing agent is required to address the mesostructured framework. Afterwards, this compound needs to be removed from the MSNs to obtain empty cavities that would host the different pharmaceuticals depending on the applications. Recently, Morales et al. [34] reported the concept of Drug-Structure-Directing Agent (DSDA), based on the use of molecules with pharmacological activity conveniently modified to be used as surfactants in the synthesis of MSNs. These DSDAs present a hydrophilic head—the biologically active molecule—and a hydrophobic hydrocarbon tail. Thus, the supramolecular chemistry of these agents in aqueous media causes the formation of micelles, which promote the formation of MSNs through electrostatic interactions with the corresponding silica precursors. Additionally, to address the MSN framework, DSDAs possess pharmacological activity and hence play a dual role that endows the MSNs synthesized by this approach with intrinsic medical attributes. The synthesis of DSDAs is carried out by amidation reactions between selected drugs and fatty acid derivatives. Following a similar approach, Stewart et al. [35] described a novel dental adhesive that includes antimicrobial drug-silica coassembled particles with antimicrobial effects.

L-arginine is a semiessential amino acid present in the diet and is also produced naturally by the body and secreted in saliva in free form or as salivary peptides [18]. L-arginine is considered a prebiotic-based biofilm modifier, as certain oral bacteria, such as *S. sanguis*, *S. parasanguinis* and *S. gordonii*, can metabolize arginine via the L-arginine deiminase pathway (ADS), producing ammonia and neutralizing the harmful acidic conditions for oral health [18, 36–39]. Moreover, different studies have demonstrated that L-arginine inhibits the growth of pathogenic bacteria, such as *S. mutans* [36, 40, 41], and its incorporation into fluorinated toothpaste has been shown to clinically reduce the incidence of dental caries [42]. Accordingly, this oral prebiotic has also been included in its free form in fluoride varnishes [43], glass ionomer cements [11], orthodontic light-cured resin cements [23] and dental adhesives [18].

**Fig. 1:**
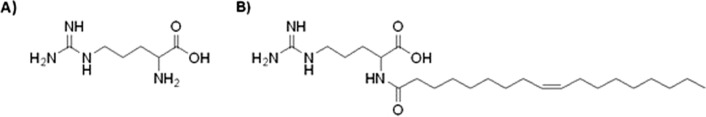
Molecular structures of **A**) L-arginine (Arg) and **B**) the N-oleoyl-L-arginine DSDA

The aim of this study is to formulate dental adhesives including different concentrations of mesoporous silica nanoparticles (MSNs) loaded with L-arginine (Arg@MSNs). The Arg@MSN drug delivery system was prepared from the DSDA approach by reaction between L-arginine (Fig. 1A) and oleoyl chloride, yielding N-oleoyl-L-arginine (Fig. 1B). The DSDA, thus prepared, formed micelle templates around which the silica sources condensed, obtaining mesoporous silica nanoparticles with all their inner porosity occupied with the DSDA (N-oleoyl-L-arginine). Subsequently, we investigated the release of DSDA containing arginine from MSNs. In vitro experiments demonstrated that L-arginine was delivered long-term in a controlled way. Furthermore, the antibacterial activity of Arg@MSNs embedded in the dental adhesive, Arg@MSN@DAdh, was investigated. It was found in the bacterial culture experiments that the adhesive system containing Arg@MSNs provided a significant reduction in the bacterial strains *Streptococcus mutans* and *Lactobacillus casei*. Finally, the incorporation of Arg@MSNs at different concentrations in the dental adhesive did not lead to any detrimental effects on the physico-mechanical and adhesive properties and anti-caries activity. Therefore, we demonstrate that the Arg@MSN drug delivery system embedded in dental adhesives (Arg@MSN@DAdh) is a very promising therapeutic approach that opens a new perspective for preventing secondary caries in an economical manufacturing way.

## Experimental

### Synthesis of the Arg@MSNs nanoparticles and the experimental adhesives

#### Chemicals

L-arginine (≥ 98%), oleoyl chloride (≥ 89%), (3-aminopropyl) triethoxysilane (APTES, 98%), tetraethyl orthosilicate (TEOS, 98%), hydrochloric acid (35% w/w), bisphenol A glycidyl methacrylate, 2-hydroxyethylmethracrylate, ethyl 4-(dimethylamino) benzoate and camphorquinone were all obtained from Sigma-Aldrich.

#### Synthesis of DSDA and N-oleoyl-L-arginine

From L-arginine (Fig. 1A), the drug structure-directing agent N-oleoyl-L-arginine (Fig. 1B) was obtained through the amidation reaction between the amino group of the asymmetric carbon of this amino acid and oleoyl chloride (C18). The reaction was performed as follows: L-arginine (1.5 g, 8.6 mmol, 1 equiv.) was dissolved in a solution of H_2_O (7 mL) and acetone (4 mL), and the mixture was stirred at a temperature in the range of 10–15 °C while adjusting the pH to 10–11 with a solution of 5 N NaOH. Then, oleyl chloride (3 mL, 9.03 mmol, 1.05 equiv.) was added dropwise to the solution over 30 min. The pH of the solution was kept between 10 and 11 by adding drops of the solution of aqueous NaOH 5 N. The reaction was stirred for 1 h, and after that, the temperature was increased to 50 °C while adjusting the pH to 3.8 with HCl (37%). Finally, when the solid was completely dissolved, the pH was adjusted to 5–6, and the final solid was filtered off and washed with water and ethanol. The solid was fully characterized by ^1^H and ^13^C NMR and ESI–MS.

#### Synthesis of mesoporous silica nanoparticles with DSDA N-oleoyl-L-arginine (Arg@MSNs)

The MSNs synthesized using DSDA N-oleoyl-L-arginine were obtained as follows. N-oleoyl-L-arginine (306 mg, 0.7 mmol, 0.5 equiv.) was dispersed in Milli-Q water (50 mL) at 90 °C overnight. The next day, the temperature was decreased to 60 °C, and (3-aminopropyl)triethoxysilane (200 µL, 0.854 mmol, 0.61 equiv) was added. The resulting mixture was stirred for 5 min before the addition of tetraethylorthosilicate (1.84 mL, 8.26 mmol, 5.90 equiv.). The pH of the mixture was adjusted to 9.5 by the addition of drops of a 1 M aqueous NaOH solution. After that, the reaction was stirred for 10 min; the stirrer was removed from the solution, and the mixture was heated overnight at 60 °C. Finally, the next day, the temperature was increased to 100 °C and maintained at that temperature for 3 days. The solid was filtered off, washed with H_2_O and ethanol, and dried under vacuum.

#### Experimental adhesive formulation

A generic dental adhesive was obtained as follows: 100 mL of bisphenol A glycidyl methacrylate (Bis-GMA) was stirred for 1 h in the presence of 48 mg of 2-hydroxyethylmethacrylate (HEMA). After that, 1.66 g of ethyl 4-(dimethylamino) benzoate and 414 g of camphorquinone were added to the previous mixture and stirred in the dark for 24 h. Arg@MSNs were added to the adhesive and homogenized at weight ratio concentrations of 0.5%, 1% and 2%, obtaining three different experimental adhesives.

### Characterization of the Arg@MSNs nanoparticles

The ^1^H and ^13^C NMR measurements were conducted on a Varian Infinity 400 MHz spectrometer fitted with a 9.4 T magnetic field. Chemical shifts (d) are shown in ppm, and they were externally referenced to tetramethylsilane (TMS). Mass measurements were performed on an ultra-high-performance liquid chromatography-tandem mass spectrometer (UHPLC-HESI-MS/MS) using a VIP Heated electrospray ionization interface (Bruker UHPLC/MSMS EVOQ^™^ ELITE) with a triple-quadrupole detector.

The MSNs were characterized by transmission electron microscopy (TEM) using a JEOL JEM 2100 microscope operating at 200 kV and with a resolution of 0.25 nm. The samples were dispersed in ethanol and deposited on a carbon-coated copper grid. The size and measurement of the nanoparticles were determined from TEM micrographs using ImageJ software. The textural properties were obtained from the N_2_ adsorption–desorption isotherms at − 196 °C using a Micromeritics TriStar 3000 instrument. Before the measurement, the samples were first calcinated at 550 °C for 5 h and then outgassed at 100 °C for 24 h with a N_2_ flux. Slit pore geometry was assumed for the calculation of the mesopore size distribution (PSD) using the NLDFT model.

FTIR analyses were collected using the attenuated total reflection technique (ATR) on a Mattson Infinity series apparatus in the wavelength range from 4000 to 600 cm^−1^ with a step size of 2 cm^−1^, and 64 scans were collected for each analysis. XRD patterns were recorded from the calcined samples on a Philips X´PERT MPD powder diffractometer equipped with CuKα radiation.

Thermogravimetric analyses (TGA) were performed under an air atmosphere with a Star system Mettler Thermobalance in the temperature range from 40 to 800 °C at 5 °C min^−1^. A NanoPlus DLS Zeta potential from Micromeritics was used to obtain the zeta potential values of the particle suspensions. The samples were suspended in Milli-Q water at a concentration of 1 mg mL^−1^ and sonicated for 30 min before measurement.

### Physico-mechanical properties of the experimental adhesives

#### Ultimate Tensile Strength (UTS)

Ten specimens (n = 10) of each of the following adhesives were prepared using 10 × 4 mm hourglass-shaped molds (Odeme Dental Research, Luzerna, SC, Brazil). They were placed into the molds and covered with a transparent Mylar strip and a glass slide.

- SB1XT: The two-step etch-and-rinse adhesive Scotchbond 1XT (3M Oral Care, Seefeld, Germany).
- SBUP: The universal adhesive Scotchbond Universal Plus (3M Oral Care).
- Unfilled: An experimental adhesive without Arg@MSNs.
- 0.5% Arg@MSNs: The same experimental adhesive was filled with Arg@MSNs at 0.5% wt.
- 1% Arg@MSNs: The same experimental adhesive was filled with Arg@MSNs at 1% wt.
- 2% Arg@MSNs: The same experimental adhesive was filled with Arg@MSNs at 2% wt.

They were all light-cured with the Elipar S10 LED unit at 1200mW/cm^2^ for 20 s (3 M Oral Care). Specimen ends were bonded to a testing jig device with cyanoacrylate glue and submitted to a tensile strength test in a universal testing machine (Instron 3345, Instron Co, Canton, MA, USA) at a crosshead speed of 0.5 mm/min. The Ultimate Tensile Strength (UTS) was calculated in MPa using the formula UTS = F/A, where F is the maximum load at fracture (N) and A is the transversal cross section area (mm^2^). The latter was determined with a digital caliper (Fino GmbH, Mangelsfeld, Germany) after fracture.

#### Flexural strength (FS) and modulus of elasticity (E)

Ten rectangular-shaped samples of each of the adhesives detailed above were prepared using silicon molds of 10 × 2 × 2 mm (Odeme Dental Research). The molds were filled with the adhesives and covered with an acetate strip and a coverslip. Then, they were light-cured for 20 s with the Elipar S10 LED unit (3 M Oral Care). Specimens were stored for 24 h at 37 °C. The width (B) and height (H) in mm of the specimens were determined for further calculations with a digital caliper (Fino GmbH). Specimens were subjected to a three-point flexural strength (FS) test in a universal testing machine (Instron 3345) with a crosshead speed of 0.5 mm/min. The maximum load at fracture (F) in N was noted, and the FS was determined with the following formula ${\text{FS}}\, = \,{\text{3FL}}/\left( {{\text{2BH}}^{{2}} } \right)$, in which L was the distance between supports. The modulus of elasticity (E) value was also calculated by using the standard equation ${\text{E}}\, = \,{\text{L3F}}/{\text{4wH3d}}$, in which L is the support span length (mm), w is the width of the specimen in mm, and d is the deflection (mm) at load F.

#### Vickers hardness (VHN)

Five disk-shaped specimens of each experimental adhesive were prepared by using 5 × 1 mm silicon molds (Odeme Dental Research). The molds were placed on a microscope slide and filled with the different adhesives that were covered with an acetate strip and pressed with a transparent glass slide to remove adhesive excess. All specimens were light-cured as previously described and removed from the molds 20 min after preparation. They were stored for 24 h in dry conditions at 37 ºC and polished with an OPA disc. Microhardness measurements were performed by means of a Vickers digital microhardness (Buehler 2101, Lake Bluff, IL, USA). Ten indentations were recorded on each specimen applying a constant load of 50 g for 30 s.

#### Degree of conversion (DC)

Five specimens of each adhesive were prepared. Each specimen was measured before and after photopolymerization for 20 s with the unit Elipar S10 Curing Light. DC was determined with a Fourier transform infrared spectrometer (Excalibur Series 3100, Varian; Walnut Creek CA, USA). The absorbance spectrum included 16 scans at a resolution of 2 cm^−1^. To analyze the uncured specimens, one drop of each adhesive was deposited on the machine's sensor and scanned. Once finished, the same sample was light-cured for 20 s and scanned again. The DC for each adhesive was calculated according to the equation ${\text{DC }}\left( \% \right)\, = \,\left\{ {{1} - \left( {{\text{Xa}}/{\text{Ya}}} \right)/\left( {{\text{Xb}}/{\text{Yb}}} \right)} \right\}\, \times \,{1}00$, where Xa (polymerized) and Xb (unpolymerized) represent the bands of the polymerizable aliphatic double bonds C = C (vinyl) (1635 cm^−1^), and Ya (polymerized) and Yb (unpolymerized) represent the bands of the aromatic double bonds C = C (1608 cm^−1^).

#### Microtensile bond strength test (µtbs)

Once approval from the Ethics Committee of Rey Juan Carlos University (Madrid, Spain) was obtained, 30 sound human third molars were selected and stored in a 0.1% thymol solution at 4 °C for up to 3 months to perform this ex vivo bonding test. The middle dentine was exposed by sectioning the crown parallel to the cementum-enamel junction with a water-cooled low-speed diamond (IsoMet 5000 Linear Precision Saw, Buehler, Lake Bluff, IL, USA). The dentin was ground with a 600-grit SiC abrasive disc (Buehler) for 60 s to create a standardized smear layer. Teeth were randomly assigned to six experimental groups, as described for the mechanical tests. All the adhesives were applied with an etch-and-rinse technique. Therefore, dentin was etched for 15 s with 36% phosphoric acid (DeTrey Conditioner 36, Dentsply Sirona), rinsed for another 15 s and air-dried. The adhesives were all actively applied by rubbing them for 20 s with a microbrush, and solvents were evaporated using a gentle air stream. The adhesives were photopolymerized for 20 s with the LED Elipar S10 Curing Light. The crowns were restored with the universal composite Ceram. X Spectra ST (Dentsply Sirona) in 3 increments of 2 mm each. Each layer was light cured for 20 s with the same curing unit. Specimens were kept in distilled water for 24 h at 37 °C. The teeth were perpendicularly sectioned in the X and Y directions with a water-cooled low-speed diamond saw (IsoMet 5000 Linear Precision Saw) to obtain beams with a cross section of 0.9 ± 0.2 mm^2^.

Each beam was individually attached to a notched stainless-steel Geraldeli's jig using cyanoacrylate glue and fractured in tension mode in an Instron 3345 universal testing machine (Instron) with a crosshead speed of 0.5 mm/min. The load at fracture was registered in N and divided by the area of each beam to calculate the microtensile bond strength values in MPa. The bonded area was determined by a digital caliper (Fino GmbH) in mm^2^.

### L-arginine delivery studies

The drug delivery study of L-arginine-containing DSDA from the Arg@MSNs was performed by suspending the nanoparticles in Milli-Q water at 37 °C with stirring. At certain times, 1 mL of the solution was removed for monitorization. After centrifugation, the concentration of DSDA in solution was obtained using a UV spectrometer (JASCO V-630) at a maximum of 196 nm. One milliliter of the removed solution was then returned to the original media directly after the UV − vis measurement.

The DSDA release experiments from the dental adhesives containing Arg@MSNs (Arg@MSNs@DAdh) were conducted as follows. Specimens of experimental Arg@MSNs@DAdh with a length of 30 mm, a width of 10 mm and a thickness of 2–3 mm were light-cured for 20 s. These specimens were cut into smaller pieces of 1–2 mm in length, width, and thickness. A total of 120 mg of these specimens was immersed in Milli-Q H_2_O with stirring at 37 °C, and aliquots were taken from 120 min to 1 week to determine the concentration of DSDA using UHPLC/MS/MS.

### Antibacterial activity of adhesives

#### Bacterial strains and culture conditions

The bacteria used in this study were *Streptococcus mutans* ATCC 25175, *Streptococcus gordonii* ATCC 33399 and *Lactobacillus casei* ATCC 393. The bacteria were kept in tubes containing Brain Heart Infusion (BHI) agar (Scharlau Chemie SA, Barcelona, Spain) at 4 °C for further use in the experiments. From the subculture of each bacterium, a 1 McFarland standard suspension was prepared in BHI broth and subsequently diluted to obtain a suspension of approximately 1 × 10^7^ colony-forming units per milliliter (CFUs/mL) [44].

#### Antibacterial activity test

The antimicrobial activity of the materials was evaluated as previously described [45, 46] by a direct contact test (DCT) with some modifications. The adhesives were inserted on the lateral wall of 96 wells of microtiter plates (Nunclon Delta Surface, Nunc, Roskilde, Denmark), one for each microbial strain. The area was delimited by two points at the edge of the wells separated by 4 mm and was coated with approximately 30 μL of each adhesive using a sterile pipette tip and a cavity liner applicator. Then, the adhesives were light-cured for 20 s, and afterwards, 10 μL of bacterial suspension was carefully placed on the surface of each adhesive. Bacterial suspensions placed on the walls of uncoated wells were used as controls.

After incubation in 100% humidity at 37 °C for 60 min, 200 μL of BHI was added to each well. Thereafter, after gently mixing with a pipette for 1 min, 100 μL of the bacterial suspension from each well was placed in Eppendorf tubes, and the well of each microtiter plate was refill with 100 μL of BHI to incubate the microbial suspension with the adhesives for a period of 7 days.

Bacterial viability was determined after 1 h and 7 days by means of the adenosine triphosphate (ATP) assay (BacTiter-Glo™, Promega, Madison, WI, USA). For the ATP assay, 100 μL of bacterial suspension was added to 100 μL BacTiter-Glow reagent in an opaque-walled 96-well plate (Greiner, Monroe, NC, USA), followed by incubation at room temperature for 5 min. The luminescence produced was measured with a luminometer (GloMax^™^, Promega) and expressed as the reduction percentage of relative light units (RLUs) in each group with respect to the control by using the following formula: $\left( {{1}\, - \,\left[ {{\text{RLUs}}_{{{\text{test}}}} /{\text{mean RLUs}}_{{{\text{control}}}} } \right]} \right)\, \times \,{1}00$ [47].

### Statistical analysis

Descriptive statistics of the values obtained in the different tests were performed using the mean as a measure of central tendency and the standard deviation as a measure of dispersion. Regarding the microhardness data, the mean of the values of each specimen was considered a statistical unit.

Data obtained in UTS, FS, E, VHN, DC and µtbs tests were analyzed using one-way ANOVA to detect statistically significant differences among experimental groups. Previously, the normal distribution of each variable was confirmed by the Shapiro–Wilk test. Post hoc comparisons were analyzed using Tukey's test.

Before statistical analysis, the data of the reduction percentage of RLUs in all groups were subjected to Anscombe transformation. Data after 1 h of contact for *S. mutans, S. gordonii* and *L. casei* determined by Shapiro–Wilk were not Gaussian, and a global comparison was performed by the Kruskal–Wallis test. Data for 7 days followed a Gaussian distribution, and there were significant differences in variances between groups by the Levene test. Global comparison was determined by ANOVA and post hoc Games-Howell test.

All statistical tests were performed at a preset alpha of 0.05 using the IBM SPSS Statistics 27 package (IBM Corporation, Armonk, NY, USA).

## Results and discussion

### Synthesis and characterization of N-oleoyl-L-arginine

A lipidic derivative of L-arginine, N-oleoyl-L-arginine, was first synthesized to act as Drug-Structure-Directing Agent DSDA, i.e., a template with pharmacological activity to drive the formation of MSNs, a concept previously introduced by us [34, 48, 49]. For this purpose, DSDA must have an amphipathic character, hydrophilic headgroup and hydrophobic tail to address the formation of micelles in water. L-arginine has several polar functional groups that can constitute the polar headgroup, so to be able to act as a DSDA, a lipidic chain must be added to the structure.

Based on these previous studies, to synthesize DSDA, L-arginine reacted with oleoyl chloride to introduce a long aliphatic chain in the molecule. Thus, *N-*oleoyl-L-arginine with amphiphilic character, polar headgroup and lipidic chain, was successfully obtained and indubitably confirmed using ^1^H and ^13^C NMR (See Additional file 1: Figure S1). The ^1^H NMR spectrum of the compound showed the signal corresponding to the proton of the asymmetric carbon of the amino acid at 4.11 ppm, which determines the formation of the amide. Moreover, all protons of the introduced oleyl chain were assigned with their corresponding integrals and ppm. The ^13^C NMR spectrum also revealed the formation of the desired product. The signals at 178 and 174 ppm correspond to the quaternary carbon of the amide group, and the split is because the spectrum was recorded below the temperature of free rotation of the amide. In addition, the signal at 157.8 ppm is typical of the carbon of the guanidine group, which indicates that there had not been a reaction between this group and oleoyl chloride. The compound was also characterized using HPLC–MS and FTIR spectroscopy (Additional file 1: Figure S2 and S3). The mass spectrum showed the correct mass of *N-*oleoyl-arginine (Additional file 1: Figure S2), and the FTIR spectrum revealed peaks that suggested the successful formation of the product. Specifically, stretching vibrations between 2950 cm^−1^ and 2850 cm^−1^ can be assigned to the -CH_2_ and -CH_3_ groups of the oleyl chain. In addition, a strong band between 1700 and 1650 cm^−1^ evidenced the stretching vibrations of the C = O bond of the secondary amide (Additional file 1: Figure S3).

The synthesized surfactant has a carboxylic acid group and a guanidinium group (Fig. 1.B). At a physiological pH, the acid would be charged negatively (pKa = 3.70) and the guanidinium positively (pKa = 12.14), so the molecule would be in a zwitterionic state with a zero net charge. In contrast, L-arginine would be charged positively at a physiological pH due to the extra amino group that has been substituted in *N-*oleoyl-arginine. To evaluate the correct formation of the product, Z potential measurements were performed in *Milli-Q* water. A value of 1.40 mV is found, very close to zero, which confirms that the DSDA surfactant is in a zwitterionic form.

### Synthesis and characterization of mesoporous silica nanoparticles with DSDAs of L-arginine (Arg@MSNs)

**Fig. 2:**
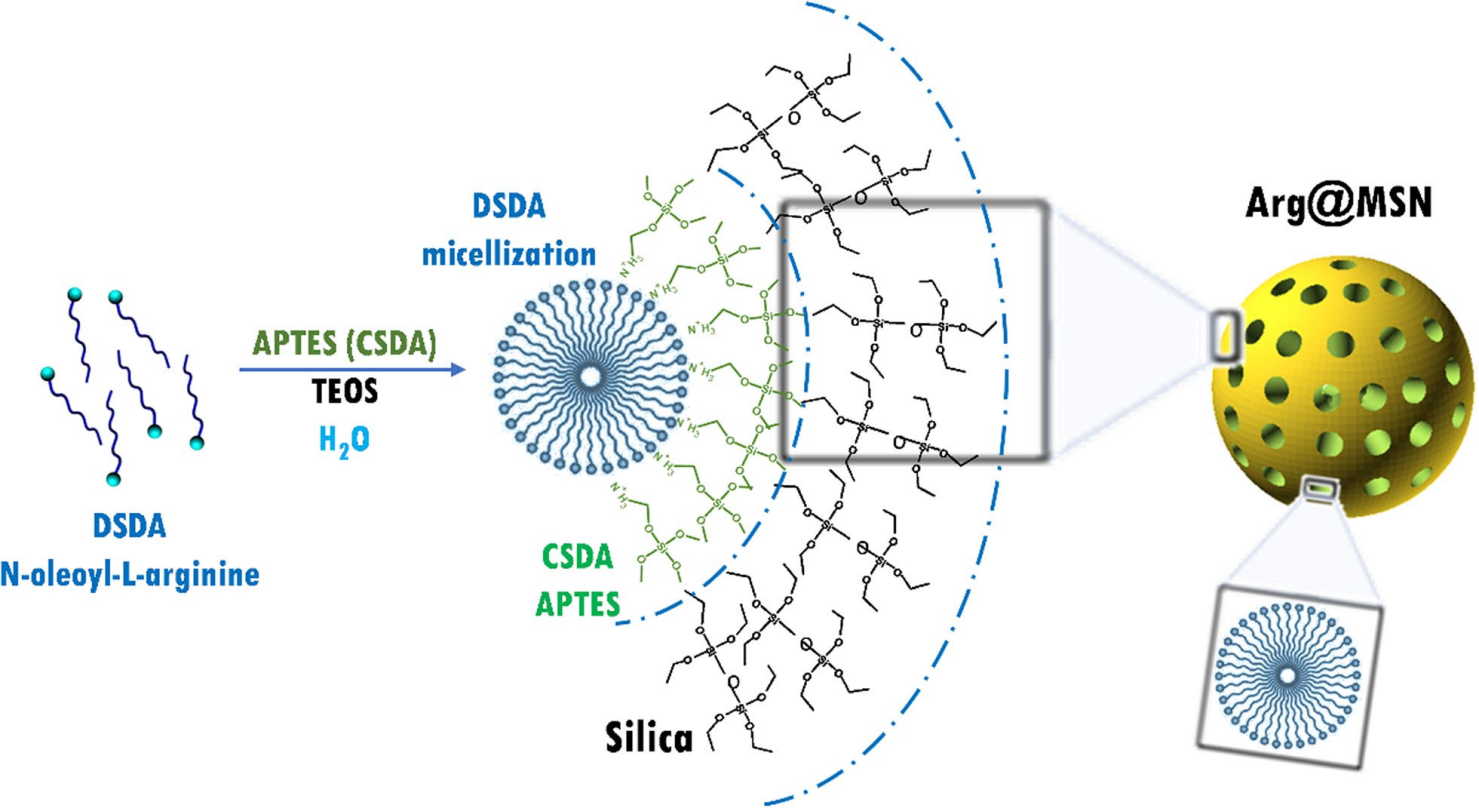
Scheme of the synthesis of Arg@MSNs using *N-*oleoyl-L-arginine as DSDA

After the synthesis and characterization of the L-arginine-containing surfactant N-oleoyl-L-arginine DSDA, the MSN nanoparticles were prepared by self-assembly with DSDA. The amphiphilic character of the DSDA molecule allows it to form micelles in contact with water. These micelles act as the template around which the silica source tetraethylortosilicate (TEOS) polymerizes via supramolecular self-assembly to create the framework of the MSNs. To neutralize the anionic form of N-oleoyl-L-arginine (DSDA) at the basic pH of the synthesis, (3-aminopropyl)triethoxysilane (APTES) was used as a co-structure directing agent (CSDA) (Fig. 2).

**Fig. 3:**
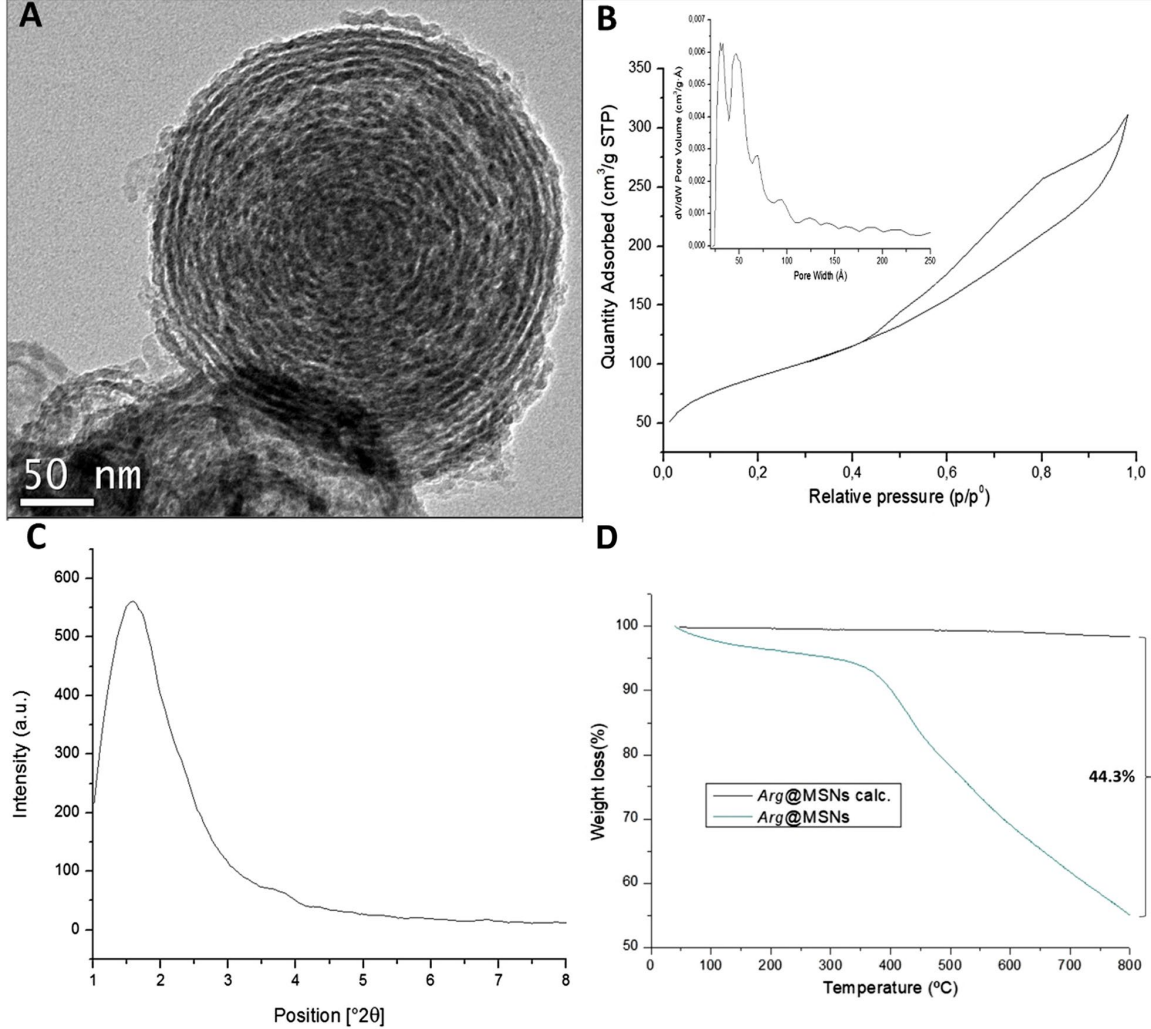
Structural and textural characterization of Arg@MSNs. TEM micrograph (**A**), N_2_ adsorption–desorption isotherm and pore size distribution (**B**), low-angle X-ray diffraction pattern (**C**) and thermogravimetric analysis (**D**)

The morphology and size of the Arg@MSNs synthesized with N-oleoyl-L-arginine as a DSDA were investigated by transition transmission electron microscopy (TEM). TEM analysis shows nanoparticles with hollow-shell and concentric spherical morphology with sizes ranging between 100 and 240 nm (Fig. 3A). Zeta potential measurements of Arg@MSNs led to a value of 1.5 mV.

**Fig. 4:**
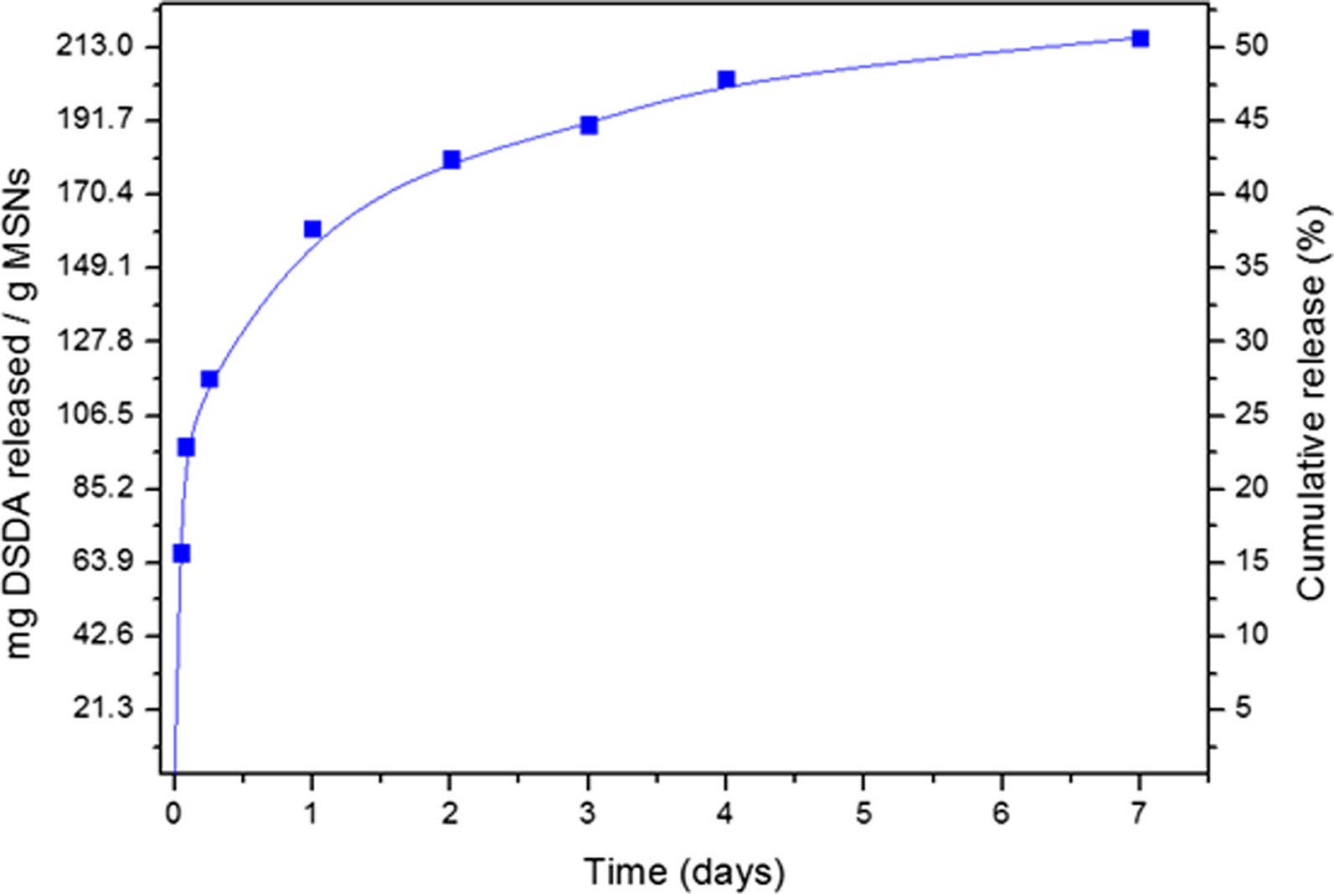
Release of the N-oleyl-L-arginine DSDA from the Arg@MSN nanoparticles

The textural properties of the nanoparticles were measured using the N_2_ adsorption–desorption experiments of the calcined material (Fig. 3B). The N_2_ isotherm presented a BET surface area of 318 m^2^ g^−1^ and a total pore volume of 0.42 cm^3^ g^−1^. The pore size distribution showed a maximum in a range between 2.2 and 5.1 nm, which revealed the morphology of mesoporous materials. The large hysteresis loop and wide pore size distribution suggest the adsorption of N_2_ molecules in the intraparticle voids that correspond with the internal hollow of these particles [34, 48, 49]. The low-angle X-ray diffraction pattern of the calcined material showed an XRD peak at 1.6 (2θ), which suggests an ordered distribution of the pores in the nanostructure of the MSNs (Fig. 4C). Figure 3D shows the thermogravimetric analysis of the Arg@MSN nanoparticles before and after calcination. TGA showed a 44.3% weight loss for the organic components (DSDA and APTES), calculated by subtracting the weight value of both samples at 800 °C.

The material was also characterized by FTIR spectroscopy (see Additional file 1: Figure S4). The spectrum showed a characteristic strong and intense band at 1037 cm^−1^, which corresponds to Si–O–Si absorption. This fact indicates that the condensation reaction between the siloxane groups of APTES and TEOS was successful. Moreover, the presence of bands corresponding to DSDA can also be observed in the spectrum, again confirming the successful incorporation into the MSNs.

### Physico-mechanical properties of the experimental dental adhesives

Then, different amounts of 0.5, 1 and 2 wt.% Arg@MSNs were incorporated into the experimental dental adhesives, their physico-mechanical properties were evaluated, and the results obtained are shown in Table 1.

**Table 1:** Mean values and standard deviation (SD) of the mechanical and physical properties of the adhesive tested

	UTS (MPa)	FS (MPa)	E (MPa)	VHN	DC (%)
*Adhesives*	*Mean (SD)*	*Mean (SD)*	*Mean (SD)*	*Mean (SD)*	*Mean (SD)*
SB1XT	12.89 (2.94) d	37.61 (15.16) de	0.483 (0.14) b	2.40 (0.55) d	26.40 (3.43) c
SBUP	61.99 (1.78) a	25.54 (5.81) e	0.581 (0.11) b	6.31 (0.68) c	33.00 (6.89) bc
Unfilled	47.90 (13.64) bc	178.5 (33.28) a	1.640 (0.29) a	11.68 (0.72) a	37.20 (4.09) ab
0.5% Arg@MSNs	40.39 (7.61) bc	109.17 (31.38) b	1.839 (0.19) a	9.84 (0.38) b	40.40 (1.14) a
1% Arg@MSNs	39.98 (13.73) c	82.15 (25.25) bc	1.707 (0.17) a	9.09 (1.44) b	36.92 (1.98) ab
2% Arg@MSNs	51.81 (4.95) ab	62.02 (16.29) cd	1.766 (0.13) a	11.44 (0.19) a	35.84 (1.06) ab

Different letters in the same column indicate statistically significant differences between the adhesive tested (p < 0.05)

In the present study, the commercial adhesives SB1XT and SBU were used as controls and references according to previous research [19]. SB1XT is a two-step etch-and-rinse adhesive that has demonstrated good performance in clinical studies [50]. SBUP is the modified version of the adhesive Scotchbond Universal, which was the first universal adhesive to be launched in the market and the one more tested [51]. Moreover, Arg@MSNs were added to a generic adhesive instead of a commercial adhesive to obtain information and control of all the ingredients included, as the full composition is not revealed by manufacturers and may affect the results [18].

In general, the values obtained for UTS, FS, E, VHN and DC with these Arg@MSN-loaded experimental dental adhesives (samples 0.5% Arg@MSNs, 1% Arg@MSNs and 2% Arg@MSNs) were higher or in the range of those determined for the two commercial adhesives tested (SB1XT and SBUP). Therefore, the experimental adhesives containing Arg@MSNs may be able to withstand the interfacial stresses due to polymerization shrinkage of the adhesive itself and the resin composite, as well as the complex masticatory forces.

Despite the adequate physico-mechanical values determined for the adhesives tested, the UTS, FS, E, VHN and DC results were not similar, and statistically significant differences were detected among them (p < 0.001). UTS is the maximum stress at which the adhesive undergoes cohesive failure. In the present study, the highest UTS values were yielded by SBUP without significant differences from those of 2% Arg@MSNs incorporated in the adhesive material. The unfilled experimental dental adhesive and the version with 0.5% Arg@MSN yielded intermediate and similar values without differences from the 1% Arg@MSNs in the adhesive. The lowest ultimate tensile strength values were obtained for SB1XT.

Regarding the FS results, the experimental unfilled adhesives yielded significantly higher values than the rest of the adhesive tested, followed by the 0.5% Arg@MSN and 1% Arg@MSN adhesives, and significantly higher values than those obtained for the 2% Arg@MSN adhesive. The higher FS values determined for the unfilled experimental adhesive can be explained by its homogeneous composition, as it mainly contains methacrylate monomers [52]. The lowest values were recorded by the commercial adhesive SBUP, without significant differences from SB1XT. This trend was also observed for the E results, as all the experimental adhesives, unfilled or charged with Arg@MSNs nanoparticles, yielded similar and significantly higher values than the commercial adhesives SB1XT and SBUP.

Therefore, increasing the amount of Arg@MSN in the generic adhesive did not affect either the UTS or E-module results. The incorporation of nanoparticles is considered to enhance the mechanical properties of dental adhesives, as they increase their viscosity, prevent the formation of thin adhesive layers, and confer radiopacity [53]. Accordingly, both commercial adhesives include silane-treated silica nanofillers in different concentrations, specifically, 10–20% for SB1XT and 5–15% for SBUP. Nevertheless, dental adhesives, unlike resin composites, cannot be highly filled to maintain their wettability. Moreover, the filler addition seems not to be the main factor influencing some mechanical properties [54], and the optimal concentration of these particles is hard to establish, as nanoparticles tend to agglomerate, creating weak points that decrease flexural strength [55]. In agreement, a trend of lower FS values was detected in our results as the amount of Arg@MSNs increased, as previously observed in other studies. [56, 57]. Similarly, in Geraldeli´s study, [18] L-arginine was included in a generic adhesive at different concentrations, detecting that as the concentration increased, UTS and FS values decreased without affecting E results.

The highest hardness values were registered by the unfilled experimental adhesive and the adhesive with 2% Arg@MSNs. Intermediate and similar hardness was obtained by the other two experimental adhesives with 0.5% and 1% Arg@MSNs. Both commercial adhesives exhibited a significantly lower hardness, with SB1XT being the softest. Accordingly, the results of the degree of conversion (DC) revealed that the four experimental adhesives obtained similar percentages of DC, with those of the 0.5% Arg@MSNs version being statistically higher than those recorded for both commercial adhesives. The lowest values were again obtained for the commercial adhesive SB1XT.

The DC of the adhesives after photopolymerization was determined by FTIR and indirectly by means of microhardness testing. The results obtained with both methodologies were similar, and they were not negatively affected by including Arg@MSNs, regardless of their concentration. The degree of conversion is considered to predict the mechanical properties of the adhesive and to influence its bonding capability [58]. According to our results, the experimental adhesives attained a degree of cure ranging from 35 to 40%. In agreement, Geraldeli et al. [18] reported similar microhardness and DC values regardless of the concentration of L-arginine included in the experimental adhesive. Moreover, the degree of conversion of our experimental adhesives in the unfilled or Arg@MSN-loaded versions was significantly higher than those of the commercial adhesives, especially SB1XT. Both SB1XT and SBU contain water (SB1XT < 5 wt% and SBUP 10–15 wt%) and ethanol (SB1XT 25–35 wt% and SBUP 10–15 wt%), unlike the nonsolvated experimental adhesives. Both components act as solvents that enhance the miscibility of the adhesive components and improve the diffusion of the resin within the dental substrate; specifically, water allows the ionization of acidic monomers included in SBUP [59]. However, they must be correctly evaporated during clinical application, as their presence may limit the degree of cure of the adhesives [59]. In our study, in the specimens prepared for VHN and DC, the adhesives were used in bulk and not in thin layers as they are used in a clinical situation. Therefore, water and ethanol may have been entrapped into the polymer with a negative impact on its DC [59].

Thus, the incorporation of Arg@MSNs at different concentrations in a generic adhesive had no deleterious effect on the physical and mechanical properties. This is of paramount relevance as the development of antibacterial properties raises concerns because they can compromise the physico-mechanical properties of the experimental dental adhesives, the bonding capacity, and their biocompatibility due to the ingredients or adhesive matrix particles leaching [18].

### Microtensile bond strength test

The mean and standard deviation values of the µtbs to dentin of the adhesives tested are shown in Table 2. Data were analysed without considering the pre-test failures. The µtbs test was chosen because it is considered one of the most standard and versatile bond strength tests that allows the evaluation of bonding ability and performance of adhesive systems. Higher bond strength values are related to a higher capacity of the adhesive interface generated to withstand stresses, including masticatory forces.

**Table 2:** Mean microtensile bond strength results in MPa and (standard deviation), percentage % of pre-test failures, and number of specimens tested of the different adhesives

Adhesives	µtbs (MPa)*	Pre-test failures (%)	Number of specimens
SB1XT	35.9 (13.9) a	23	67
SBUP	36.4 (18.7) a	31	60
Unfilled	20.0 (9.3) bc	37	61
0.5%Arg@MSNs	22.6 (9.6) b	32	64
1%Arg@MSNs	20.9 (10.3) bc	59	39
2%Arg@MSNs	15.3 (7.5) c	52	43

* Different letters in the same column indicate statistically significant differences between the adhesives tested (p < 0.05)

In the present study, the highest values were determined when the resin composite was bonded with both commercial adhesives, SB1XT and SBUP. SBUP, a universal adhesive, can be used in etch-and-rinse, self-etch or self-etch with selective enamel etching mode [51], and the adhesive strategy that provides a better clinical performance [60]. In the present study, it was used after etching the dentin with phosphoric acid, as well as the experimental adhesives, to standardize the adhesive strategy based on complete smear layer removal.

Lower and similar values were yielded by the experimental adhesives without Arg@MSNs or in a concentration of 0.5% and 1%. The lowest results were determined for the 2% Arg@MSN experimental adhesive, without significant differences from the unfilled version of the experimental adhesive and the one with 1% Arg@MSNs.

Therefore, the incorporation of L-arginine in loaded MSNs allowed adequate bond strength values to be attained, in contrast to the results reported by [18], who included free arginine at 5, 7 and 10% in a generic etch-and-rinse adhesive and obtained low microtensile bond strength values [18]. Although nanofillers are considered to increase the mechanical properties of the adhesive layer, helping to distribute the stresses due to adhesive and resin composite polymerization shrinkage, this positive effect was not observed in our study. In contrast, a detrimental effect on the microtensile bond strength was observed when the amount of the particles was increased up to 2% by weight, as observed in the FS results. Nanoparticles at high concentrations may agglomerate, hampering diffusion in the etched dentin and dentinal tubules, in agreement with Azad et al., [55]. Other authors have attributed the limited adhesive properties of experimental adhesives with nanofillers to a reduction in the degree of cure that affects their mechanical properties [20]. However, in the present study, this relationship was not observed, as DC and VHN values were higher for the filled experimental adhesives than those determined for the commercial adhesives, which yielded the highest bond strength values. The microtensile bond strength of the adhesives seems to be more related to E values than to other mechanical properties [56], and accordingly, the experimental adhesives exhibited a distinct and more brittle behaviour than SB1XT and SBUP.

### L-arginine release studies

The release of the N-oleyl-L-arginine DSDA from the Arg@MSNs was investigated by sinking the material in Milli-Q water at 37 °C and calculating the concentration in solution at different times. The mg of DSDA released per gram of MSN was plotted against time to construct the profile of the release curve (Fig. 4). The N-oleyl-L-arginine DSDA release from MSN material showed a slow and maintained delivery over several days up to approximately 210 mg of DSDA per gram of MSNs. After 6 h, the MSN material released 110 mg of DSDA, which corresponds to approximately 25% of the maximum theoretical release of the material. After 2 days, the amount increased to 180 mg of DSDA and approximately 40% of the cumulative release. The DSDA continued being released from MSN after 7 days when the release stayed at approximately 50% of the theoretical maximum release, without reaching the plateau. It can be concluded that the MSNs permit a slow and long-term release of DSDA, and the drug travels from the inside of the nanoparticle porosity to the media.

**Fig. 5:**
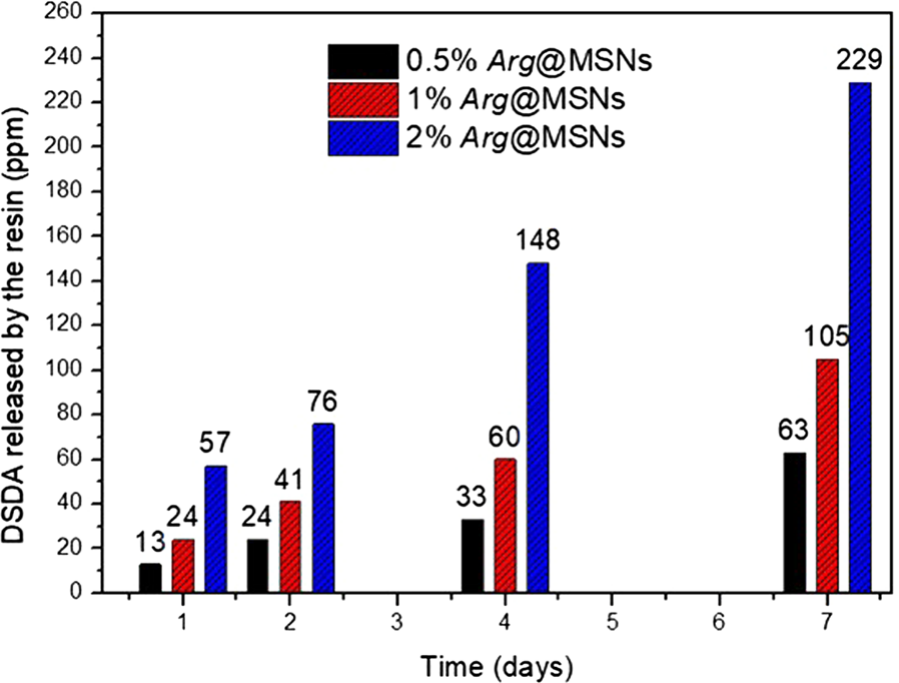
Release of the DSDA of L-arginine from the experimental adhesives with concentrations of 0.5 wt% (black), 1 wt% (red) and 2 wt% (blue) *Arg*@MSNs

Subsequently, after embedding different amounts of Arg@MSN into the dental adhesives, the release of the N-oleyl-L-arginine DSDA from the adhesive matrix was studied. The three different resins with amounts of 0.5%, 1% and 2% Arg@MSN were suspended in milli-Q water at 37 °C. Aliquots were taken at 1, 2, 4 and 7 days to calculate the amount of DSDA released from the resins and determined by UHPLC/MS/MS. Figure 5 shows that the experimental dental adhesive with 0.5% Arg@MSN released 13 ppm after 1 day, 24 ppm at 2 days and 33 and 63 ppm DSDA at 4 and 7 days, respectively. The amount of DSDA released increased accordingly as the 1% and 2% adhesives were tested in the release experiments. The 1% dental adhesive experienced a release of 24 ppm DSDA at 1 day and 60 ppm and 105 ppm at 4 and 7 days, respectively. The 2 wt.% adhesive released concentrations of 57, 148 and 229 ppm DSDA after 1, 4 and 7 days, respectively. These results indicate the ability of N-oleyl-L-arginine DSDA to diffuse from inside the nanoparticle to the resin and finally from there to the aqueous medium. Therefore, the polymeric adhesive matrix not only allows the release of N-oleyl-L-arginine DSDA but also significantly slows its delivery, which significantly lengthens the antibacterial activity provided by L-arginine over time.

### Antibacterial activity

**Fig. 6:**
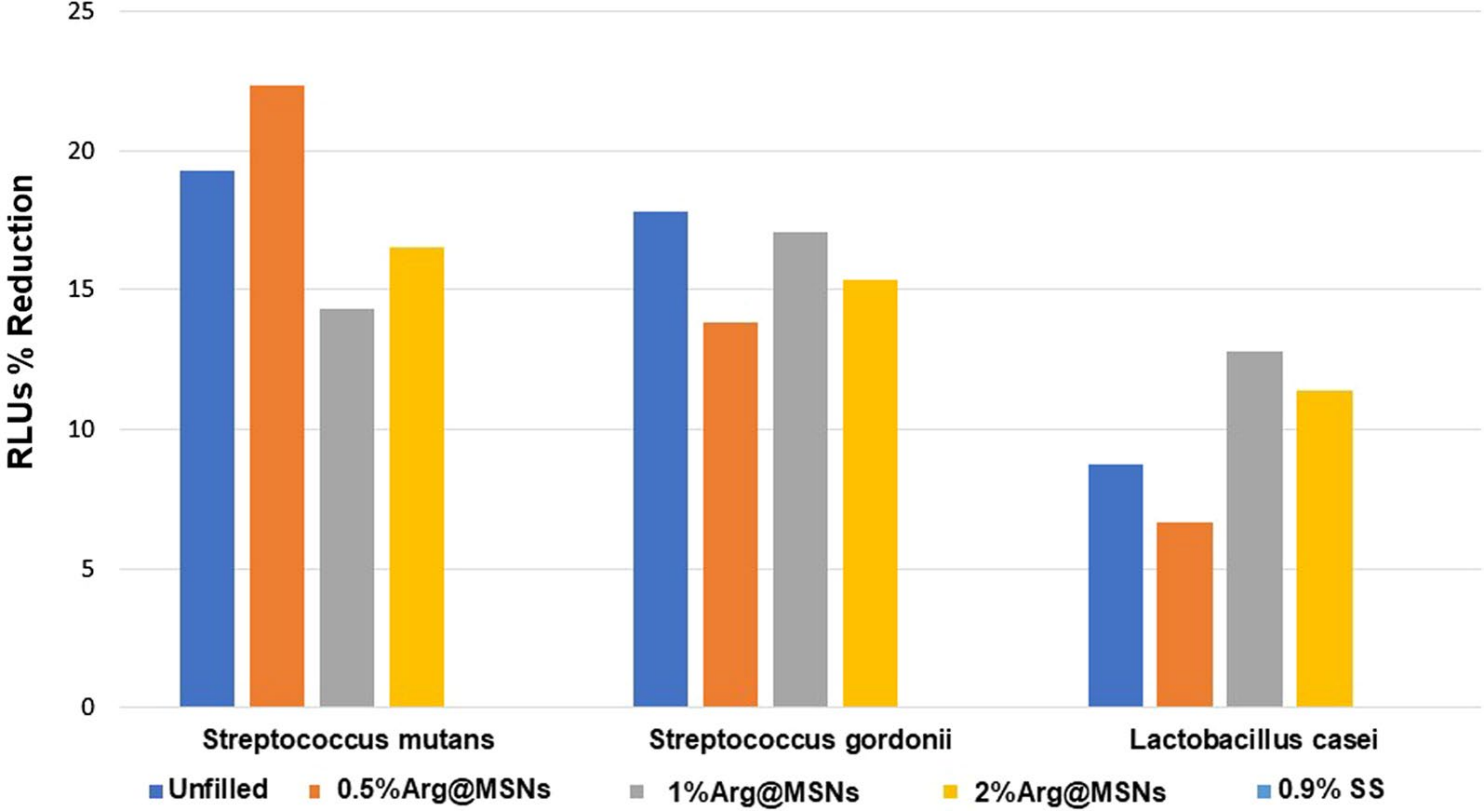
Mean reduction percentage of RLUs with the ATP assay after 1 h of adhesive contact with the bacteria. n = 16/group (Mean values of RLUs control: *S. mutans* 2167; *S. gordonii* 2426; *L. casei:2022*.)

**Fig. 7:**
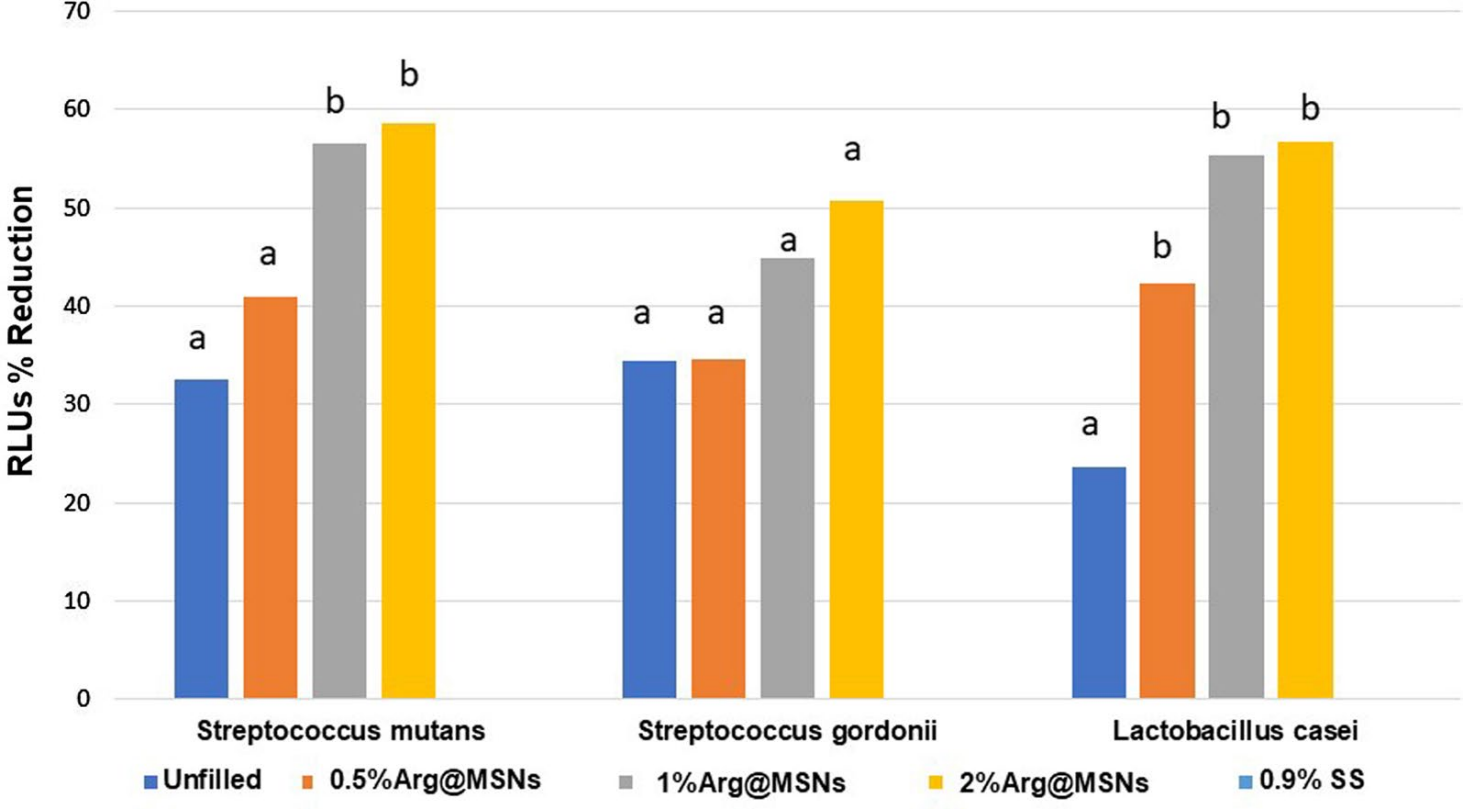
Mean reduction percentage of RLUs with the ATP assay after 7 days of adhesive contact with the bacteria. n = 16/group. (The same letters indicate no differences between experimental adhesives for each bacterial strain (P < 0.05). Mean values of RLUs control: *S. mutans* 5524; *S. gordonii* 5246; *L. casei*: 6131)

The results of the reduction percentage of RLUs are shown in Tables 3 and 4 and Figs. 6 and 7 for each bacterial strain. At 1 h, there were no significant differences between groups, *S. mutans (p* = *0.603), S. gordonii (p* = *0.326) and L. casei (p* = *0.438)*. After 7 days, the highest percentage reduction of RLUs was obtained for the 2% Arg@MSN adhesive version. The trend in all cases was for increased antimicrobial activity with higher nanoparticle concentration, although it should be noted that there were no significant differences between the experimental groups for *S. gordonii*, nor for the unfilled dental adhesive and 0.5% Arg@MSNs version for *S. mutans*. However, there were significant differences for all groups with Arg@MSNs compared to the unfilled version for *L. casei*. In 2017, Geraldeli et al. [18] proposed the inclusion of L-arginine at a concentration of 7% in a dental adhesive, reporting that its release endowed a significant antibacterial effect against *S. mutans* that was specific for this cariogenic bacterium. This behavior as a biofilm modifier was also observed when 4% L-arginine was added to a glass ionomer cement [11] and a commercial orthodontic resin cement [23].

**Table 3:** Mean and (standard deviation) of the reduction percentage of RLUs with the ATP assay after 1 h of adhesive contact with the bacteria

Adhesives	RLUs % Reduction*		
	Streptococcus mutans**	Streptococcus gordonii**	Lactobacillus casei**
Unfilled	19.30 (29.10)	17.80 (27.81)	8.74 (13.36)
0.5% Arg@MSNs	22.36 (32.97)	13.82 (25.13)	6.65 (10.96)
1% Arg@MSNs	14.30 (26.59)	17.10 (26.10)	12.77(12.39)
2% Arg@MSNs	16.50 (24.46)	15.36 (21.40)	11.41 (17.78)
0.9% SS^#^	–	–	–

^*^Values of RLUs control: mean (standard deviation): *S. mutans* 2167(426); *S. gordonii* 2426 (603); *L. casei 2022* (234)

^**^Global comparison between groups determined Kruskal–Wallis: *S. mutans (P* = *.603); S. gordonii (P* = *.326); L. casei (P* = *.438)*

^**#**^SS saline solution. RLUs: relative light units

Mean (SD). n = 16/group

**Table 4:** Mean and (standard deviation) of the reduction percentage of RLUs with the ATP assay after 7 days of adhesive contact with the bacteria

Adhesives	RLUs % Reduction*		
	Streptococcus mutans**	Streptococcus gordonii**	Lactobacillus casei**
Unfilled	32.56 (18.59)a	34.47 (27.16)a	23.58 (24.02)a
0.5%Arg@MSNs	40.98 (17.53)a	34.66 (26.30)a	42.36 (21.76)b
1%Arg@MSNs	56.52 (11.11)b	44.85 (24.65)a	55.32 (20.98)b
2%Arg@MSNs	58.59 (8.83)b	50.66 (31.16)a	56.73 (25.10)b
0.9% SS^#^	–	–	–

^*^Values of RLUs control: mean (standard deviation): S. mutans 5524(1886); S. gordonii 5246 (303); L. casei 6131(1648)

^**^Global comparison between groups determined by ANOVA (P < .001) with Welch´s correction. The same letter read vertically indicates differences that were not statistically significant according to the Games-Howell test

^**#**^*SS* saline solution, *RLUs* relative light units

Mean (SD). n = 16/group

In the present study, a different approach is evaluated. L-arginine was incorporated into MSNs, resulting in Arg@MSN nanoparticles, and in turn, these drug delivery systems were embedded in different concentrations, for the first time as far as we know, in an experimental dental adhesive. This Arg@MSN@Dadh system provides adequate and sustained antibacterial activity over time, acting as a biofilm modifier as only cariogenic bacteria were suppressed. This effect was not evident immediately; however, a significant reduction in the percentage of RLUs for *S. mutans* and *L. casei* was observed after 7 days. The antibacterial activity against *L. casei* was observed even with the lowest concentration of Arg@MSNs. Nevertheless, in the case of *S. mutans*, 1 wt.% Arg@MSNs was required to obtain a significant reduction in the percentage of RLUs. Moreover, a slightly higher antibacterial effect was detected when the concentration of Arg@MSNs was increased to 2 wt.%. Moreover, the percentage of RLUs of *S. gordonii*, the oral microbes responsible for the pH-raising effect of the oral biofilm, was not affected by the inclusion of Arg@MSNs in the experimental adhesives, which is a good result.

The inclusion of a natural amino acid, such as L-arginine, instead of other antibacterial agents limits the possibility of antimicrobial resistance in pathogens [61] while promoting the growth of healthy bacteria that may maintain or restore homeostasis in biofilms adjacent to resin composite restorations [11]. This could inhibit or limit the progression of secondary caries lesions.

The unfilled dental adhesive also showed antibacterial potential, although more limited than in the case of the Arg@MSN@DAdh system, which could be attributed to the antibacterial activity of the unpolymerized components of the adhesive, which are toxic to bacteria [62].

This is the first study to describe the development and characterization of an etch-and-rinse adhesive system containing Arg@MSNs that provides antibacterial activity without compromising the physico-mechanical and bonding properties. L-arginine incorporated into dental materials opens a new perspective for the prevention of secondary caries in a cost-effective way. Further long-term studies are required to check the longevity of the resin-dentin interface of these Arg@MSN-filled adhesives.

## Conclusions

We have developed a new drug delivery system, Arg@MSN@DAdh, for the treatment and prevention of secondary caries. The inclusion of Arg@MSNs at 0.5 wt.%, 1 wt.% and 2 wt.% in a generic adhesive did not affect their physico-mechanical and bonding properties, being able to deliver L-arginine in a sustained way with a long-term antibacterial activity enough to selectively inhibit the growth of cariogenic bacteria such as *S. mutans* and *L. casei*., and hence preventing biofilm formation over the adhesive surface. The release of L-arginine-containing DSDA was proven through in vitro experiments to be mediated by progressive diffusion over time from the internal mesoporosity of the MSNs towards the resin compound in contact with the aqueous medium. Ex vivo studies showed that the incorporation of MSN particles containing DSDA with L-arginine into the dental adhesive did not affect either its bond strength or its degradation by polymerization shrinkage. Therefore, it has been demonstrated that these MSN-dental adhesive hybrid systems that maintain the mechanical and adhesive properties of resin composites, together with a long-term release with antibacterial effects, may have enormous potential in dental caries restoration.

## Supplementary Information

**Additional file 1: Figure S1.** ^1^H NMR and ^13^C NMR spectra of the DSDA N-oleoyl-arginine in CDCl_3_. **Figure S2.** ESI-Mass spectrum of the DSDA N-oleoyl-L-arginine. **Figure S3.** FTIR spectrum of the DSDA N-oleoyl-L-arginine. **Figure S4.** FTIR spectrum of the Arg@MSNs.

## Data Availability

Not applicable.
