# Supplementary material for: L-arginine-containing mesoporous silica nanoparticles embedded in dental adhesive (Arg@MSN@DAdh) for targeting cariogenic bacteria
Source: J Nanobiotechnology. 2022 Dec 1;20:502. doi: 10.1186/s12951-022-01714-0 (PMC9714087; doi:10.1186/s12951-022-01714-0)
Supplement: Supplementary file 1 — Additional file 1: Figure S1. 1H NMR and 13C NMR spectra of the DSDA N-oleoyl-arginine in CDCl3. Figure S2. ESI-Mass spectrum of the DSDA N-oleoyl-L-arginine. Figure S3. FTIR spectrum of the DSDA N-oleoyl-L-arginine. Figure S4. FTIR spectrum of the Arg@MSNs. [file 12951_2022_1714_MOESM1_ESM.docx]

**Additional file**

**L-Arginine-containing mesoporous silica nanoparticles embedded in dental adhesive for targeting cariogenic dental biofilm**

^1^Marta López Ruiz, ^1^Victoria Fuentes Fuentes, ^1^Isabel Giráldez de Luis, ^1^Laura Ceballos García*, ^2^Carmen María Ferrer Luque, ^3^Paloma Fernández García, ^3^Samuel Martínez-Erro, ^3^Francisco Navas López, ^3^Victoria Morales Pérez, ^3^Raúl Sanz Martín, ^3^Rafael A. García-Muñoz*

^1^Faculty of Health Sciences, IDIBO Research Group, Rey Juan Carlos University, Madrid, Spain

^2^Department of Stomatology, School of Dentistry, University of Granada, Campus de Cartuja, Colegio Máximo s/n, E-18071, Granada, Spain

^3^Department of Chemical and Environmental Technology, Rey Juan Carlos University, C/ Tulipán s/n, 28933, Móstoles, Madrid, Spain

*E-mail: [rafael.garcia@urjc.es](mailto:rafael.garcia@urjc.es)

[laura.ceballos@urjc.es](mailto:laura.ceballos@urjc.es)

DSDA N-oleoyl-arginine (N-oleoyl-arginine)

**^1^H NMR (CDCl_3_):** δ 5.32 (m, 2H (H_12_, H_13_)); 4.11 (m, 1H (H_4_)); 3.17 (m, 2H (H_1_)); 2.20 (m, 2H (H_5_)); 2.00 (m, 4H (H_11_, H_14_)); 1.74 (m, 2H (H_20_)); 1.56 (m, 4H (H_6_, H_10_)); 1.24 (s, 20H (H_2_, H_3_ H_7_, H_8_, H_9_, H_15_ ,H_16_, H_17_ ,H_18_, H_19_)); 0.87 (t, 3H, J = 7.2 Hz (H_21_)). **^13^C RMN (CDCl_3_):** δ 180.5 (C_f_), 178.1 (C_g_), 174.3 (C_g_), 157.8 (C_a_), 130.2 (C_o_), 129.8 (C_p_), 54.4 (C_e_), 41.0 (C_b_), 36.8 (C_h_), 32.1 (C_v_), 30.1, 30.0, 29.8, 29.6 (C_d_, C_j_, C_k_, C_l_, C_m_, C_n_, C_q_, C_r_, C_s_, C_t,_ C_u_), 27.5 (C_c_), 26.2 (C_i_), 22.9 (C_w_), 14.3 (C_x_). **UHPLC/MS [M+H]^+^** = 439.4.


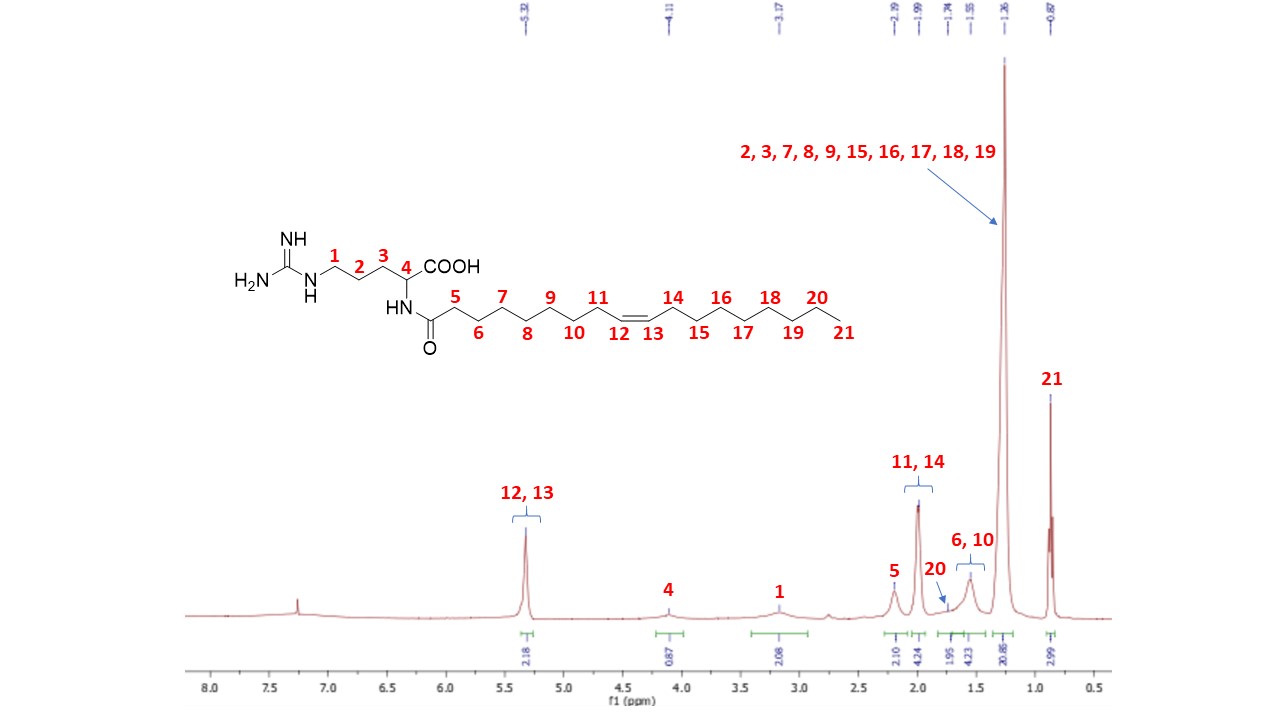


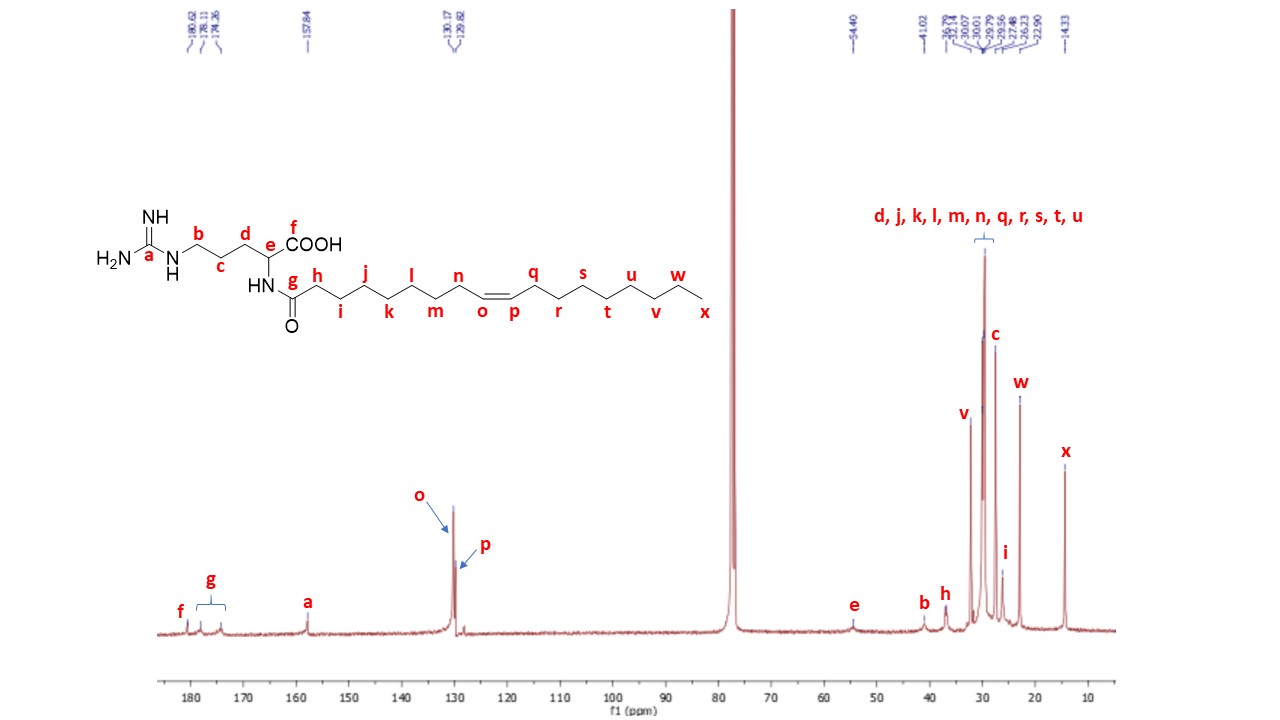


**Figure S1.** ^1^H NMR and ^13^C NMR spectra of the DSDA *N-oleoyl-arginine* in CDCl_3_*_._*


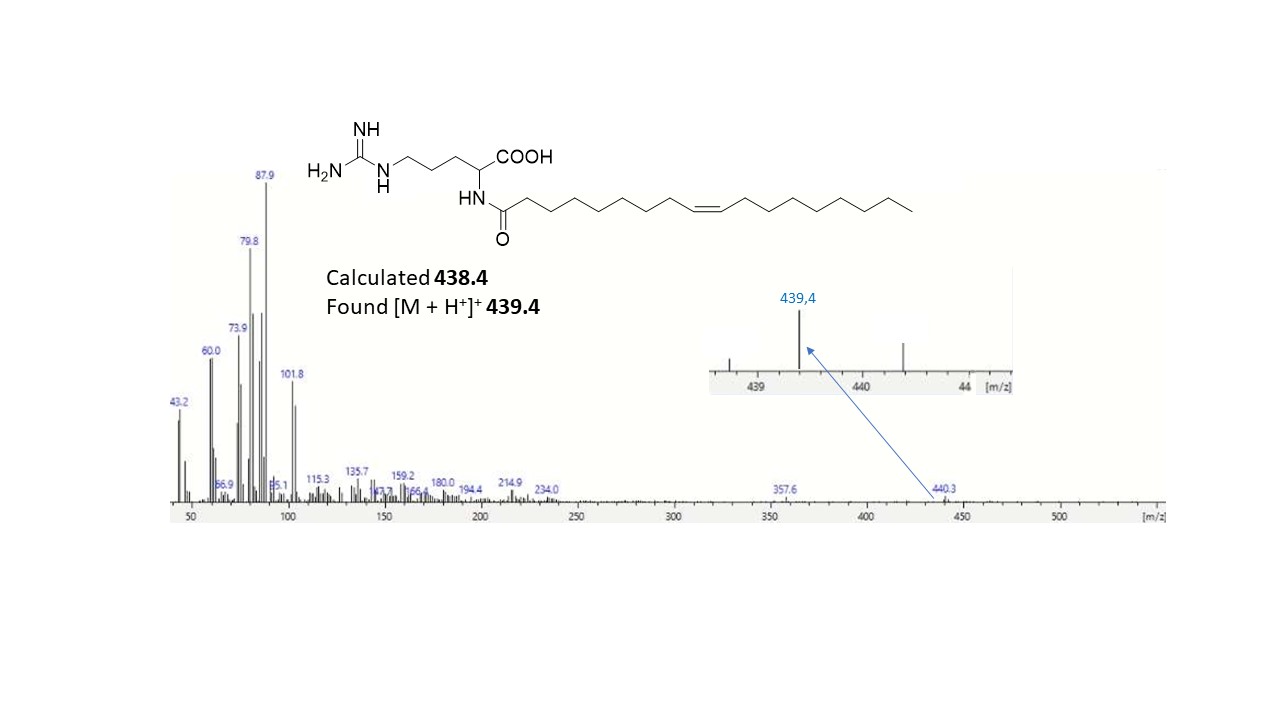


**Figure S2.** ESI-Mass spectrum of the DSDA *N-oleoyl-arginine_._*


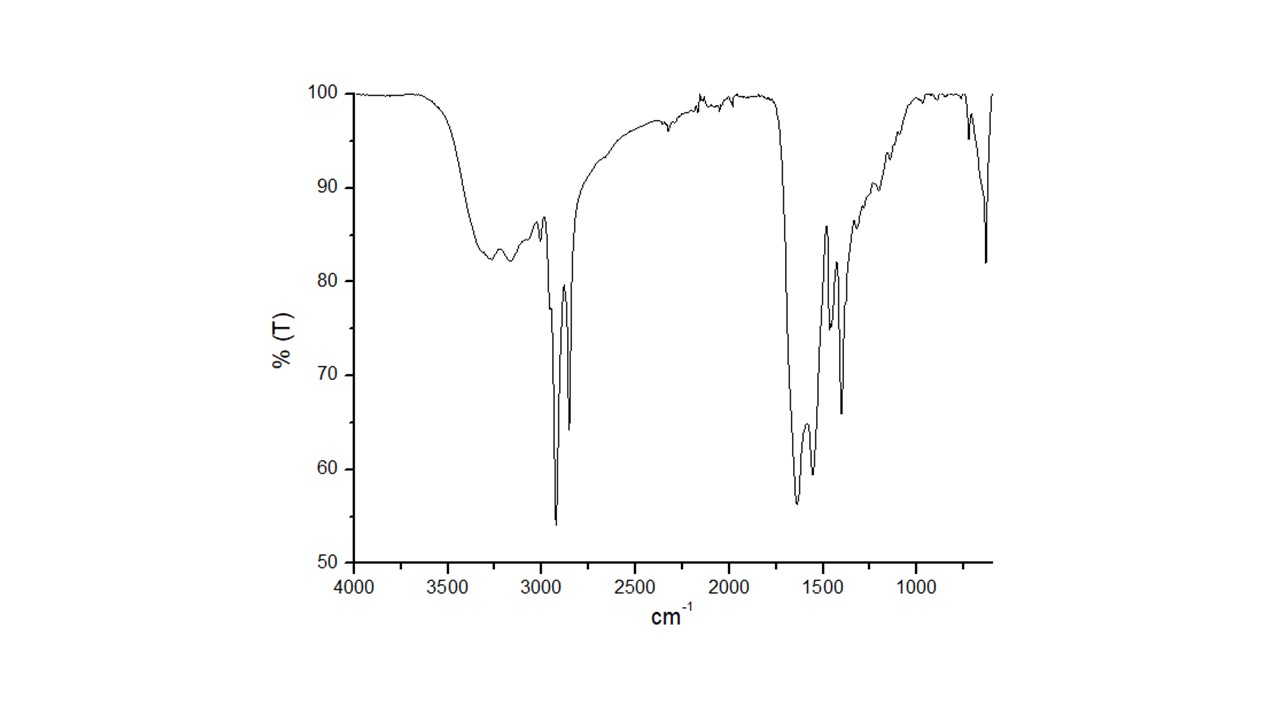


**Figure S3.** FTIR spectrum of the DSDA *N-oleoyl-arginine_._*


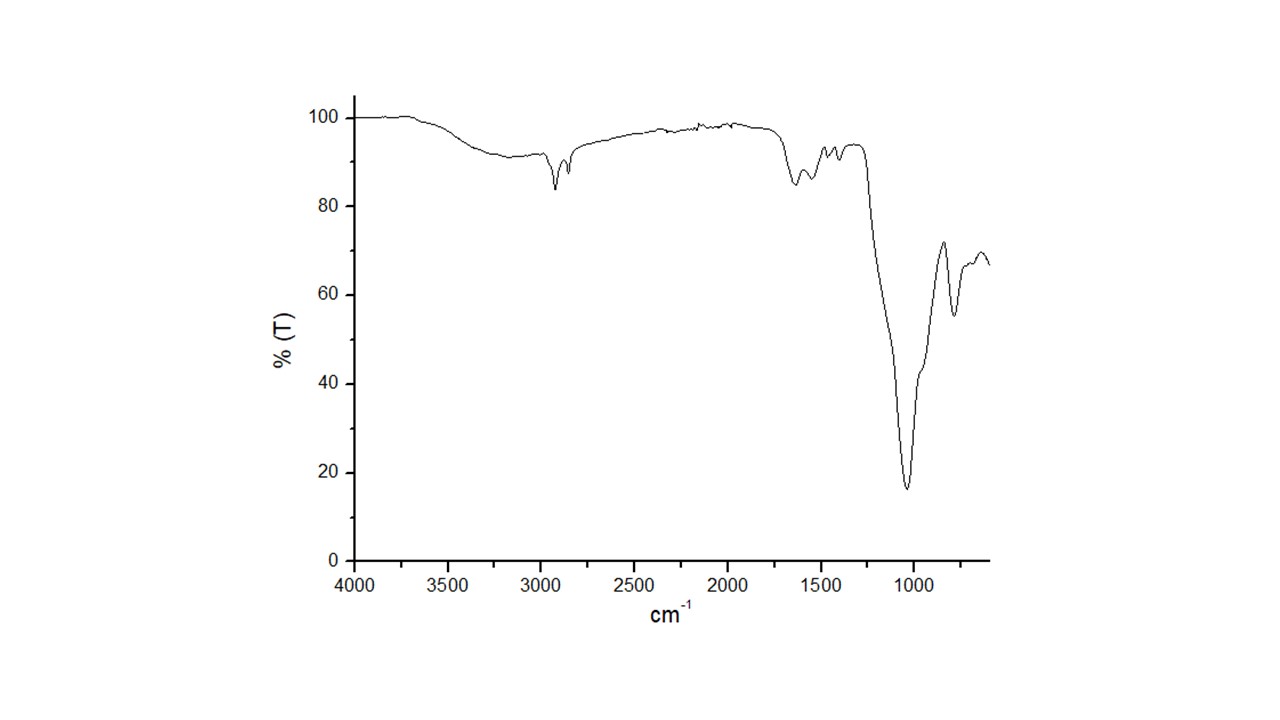


**Figure S4.** FTIR spectrum of the *Arg@MSNs.*
